# CRISPR-Based Genetic Switches and Other Complex Circuits: Research and Application

**DOI:** 10.3390/life11111255

**Published:** 2021-11-17

**Authors:** Pei Du, Chunbo Lou, Xuejin Zhao, Qihui Wang, Xiangyu Ji, Weijia Wei

**Affiliations:** 1 CAS Key Laboratory of Pathogen Microbiology and Immunology, Institute of Microbiology, Chinese Academy of Sciences, Beijing 100101, China; zhaoxj@im.ac.cn (X.Z.); wangqihui@im.ac.cn (Q.W.); 2 CAS Key Laboratory of Quantitative Engineering Biology, Shenzhen Institute of Synthetic Biology, Shenzhen Institutes of Advanced Technology, Chinese Academy of Sciences, 1068 Xueyuan Avenue, University Town, Nanshan, Shenzhen 518055, China; cb.lou@siat.ac.cn; 3 CAS Key Laboratory of Microbial Physiological and Metabolic Engineering, Institute of Microbiology, Chinese Academy of Sciences, Beijing 100101, China; jixiangyu14@mails.ucas.edu.cn (X.J.); weiweijia15@mails.ucas.ac.cn (W.W.); 4 College of Life Science, University of Chinese Academy of Sciences, Beijing 100049, China

**Keywords:** CRISPR, genetic switch, transcription, translation, genetic circuit

## Abstract

CRISPR-based enzymes have offered a unique capability to the design of genetic switches, with advantages in designability, modularity and orthogonality. CRISPR-based genetic switches operate on multiple levels of life, including transcription and translation. In both prokaryotic and eukaryotic cells, deactivated CRISPR endonuclease and endoribonuclease have served in genetic switches for activating or repressing gene expression, at both transcriptional and translational levels. With these genetic switches, more complex circuits have been assembled to achieve sophisticated functions including inducible switches, non-linear response and logical biocomputation. As more CRISPR enzymes continue to be excavated, CRISPR-based genetic switches will be used in a much wider range of applications.

## 1. Introduction

As a fast-growing multidisciplinary field, synthetic biology has aimed to revolutionize biological research with the concept of engineering since its earliest days. In two decades, numerous synthetic parts with increasing complexity have emerged with the capability of mimicking the function of a number of basic electric circuits, including biological bistable switches [1], oscillators [2], spatial pattern formation [3,4], logic gates [5,6,7], memory devices [8,9], intercellular communication [10,11,12,13] and so on. Built upon these parts, synthetic biological circuits have been introduced into a wide range of applications including autonomous metabolic engineering [14,15,16,17], cell-based therapies [18], antibiotic-free pathogen control [19,20] and so on. These circuits operate on all levels of life including transcription, translation and post-translation levels. Nevertheless, at their core, the essence of synthetic circuits is to exert precise spatial and temporal control of gene expression as output signals in response to input signals.

Compared to the traditional gene regulation elements such as inducible promoters, synthetic circuits are far superior because of their complexity that enables a sensitive or adaptative response, multiple inputs/outputs and logical signal computation. However, attempts to further increase the complexity of genetic circuits have been hindered by the inherent properties of basic synthetic parts. For example, transcription factors (TFs), the most widely adopted gene expression regulator in circuits, are known to be challenging from an engineering perspective. Specifically, the TFs offer important characteristics such as a high dynamic range and ultrasensitive response to the regulation of gene expression, but engineering a TF with specific parameters is a difficult task because of (1) poor designability, (2) a limited choice of TFs and (3) a physiological burden to host. These unsolved problems urge the development of modular, orthogonal and easily programmable synthetic parts to enable the building of more sophisticated genetic circuits.

In recent years, Clustered Regularly Interspaced Short Palindromic Repeats (CRISPR) systems have shed light on these problems by offering a unique category of synthetic parts, which are particularly useful for building complex genetic circuits. The CRISPR systems are natural defense mechanisms in bacteria and archaea for inactivating foreign nucleic acids [21]. Basically, CRISPR enzymes identify exogenous nucleic acids and record short fragments of them in the CRISPR array of the host genome as “immune memory”. Then, the short fragments (often referred to as “spacers”), along with repeat elements separating them, are transcribed as pre-crRNA, which is subsequently processed by specific endoribonucleases into “guide” RNAs (gRNA) for directing CRISPR endonucleases to the invading nucleic acids. With such “memory”, subsequent invasion by recognizable foreign DNA (or RNA) will be detected by the “guide” and inactivated by endonucleases.

CRISPR endonucleases are particularly suitable to be engineered as genetic switches because of their abundance, orthogonality and programmability. First, CRISPR systems are widely discovered in bacteria and archaea, which bestows an enormous pool of enzymes with diverse functions capable of targeting both DNA and RNA [22]. Compared to the protein-DNA binding of TFs, the fact that CRISPR endonuclease utilize a highly programmable oligonucleotide to recognize DNA makes it much more specific, reliable and friendly to standardize in in silico design. The nearly infinite number of possible oligonucleotide sequences also offers unrivaled potential for orthogonality. More importantly, conventional genetic switches, such as the TF-based switches, operate only on a transcription level, but the diverse nature of CRISPR systems allows the development of genetic switches that control gene expression through both transcription and translation, which greatly expands the territory of gene regulation. Therefore, by harnessing these unique features, a large number of highly modular and orthogonal CRISPR-based genetic switches have been developed in recent years (Table 1).

## 2. CRISPR-Based Genetic Switches in Transcription Level

The first widely used CRISPR-based genetic switches functioning at transcription level are the CRISPR/dCas9 systems designed based on the Cas9 endonuclease, an RNA-guided DNA endonuclease originating from subtype II of the class 2 CRISPR system [57]. As a CRISPR endonuclease, Cas9 identifies its target through a short PAM (protospacer adjacent motif, NGG in *Streptococcus pyogenes*), binds to the adjacent 20 nt dsDNA sequence through two RNA duplexes and cleaves at a specific location [58]. Such specificity and programmability make Cas9 an ideal candidate as a tool for gene editing and gene expression regulation. For simplicity and convenience, the two RNA duplexes for Cas9 were combined as a single guide RNA (sgRNA) [59]. In addition, although the typical length of dsDNA binding region within a sgRNA is 20 nt, shortening such a length to 17–18 nt could benefit the on-target cleavage efficiency of Cas9 [60].

**Figure 1:**
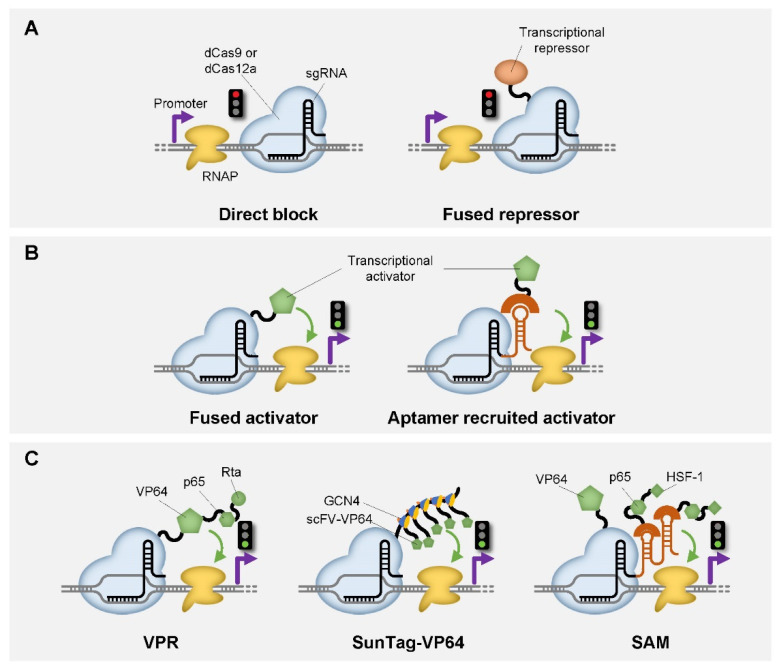
Schematic representation of CRISPR-based genetic switches in transcription level. (**A**) Schematic of dCas9 or dCas12a function as a transcriptional repressor by blocking RNAP by itself (Left), with a fused repressor such as Mxi1, KRAB or SRDX domain (Right). (**B**) Schematic of dCas9 or dCas12a function as a transcription activator by fusing with an activation domain such as AsiA and the RNAP ω (omega) subunit, VP64, VPR, etc. (left), or by recruiting an activator through the aptamer fused with sgRNA such as SoxS and PspF (Right). (**C**) Schematic of dCas9 or dCas12a function as a transcriptional activator in combination with various types of activation domains. Red light indicates repression, green light indicates activation.

To engineer endonucleases as a part of genetic switches, the endonuclease activity of these enzymes was deactivated, leaving only the DNA binding activity. Take Cas9 as an example. Two mutations (D10A and H840A) were introduced to the RuvC and HNH endonuclease domain of SpCas9 (Cas9 from *Streptococcus pyogenes*), which abolished its ability to cleave dsDNA. The resulting deactivated Cas9, with the capability to tightly bind to DNA, was named dCas9 and often used as a programmable “roadblock” of transcription in genetic switches [23] (Figure 1A, left). Such a “roadblock” can serve as a transcriptional repressor, which is often referred to as CRISPR interference (CRISPRi). Therefore, genetic switches based on CRISPRi would function as a NOT gate, which generates an output decreased signal as the input signal increases. In recent years, dCas9 has been successfully demonstrated as CRISPRi alone in bacteria [23,24], alone or with fused additional Mxi1 domain in yeast [26], and a fused KRAB (Krüppel associated box) [25,26,27,28,29] or SRDX (EAR-repression) [30] domain in mammalian cells and plants, respectively (Figure 1A, right). On the other hand, dCas9 can also be engineered as a transcriptional activator (CRISPRa), by incorporating an activation domain by either direct fusion, RNA-scaffold recruitment, or the combination of both approaches (Figure 1B). For prokaryotic cells, the activation domains available to be directly fused to dCas9 include the phage activator AsiA and the RNAP ω (omega) subunit [24,44]. Some other activation domains, including SoxS and PspF, can be fused to RNA binding domains and indirectly recruited by dCas9 through binding with the RNA scaffolds connected to sgRNA [45,46,47]. Interestingly, the output of transcription regulation can be tuned by manipulating the DNA binding region of sgRNA by introducing mismatches [47]. In eukaryotic cells, all the reported dCas9-based transcriptional activators are designed by direct fusion of an activation domain, including multiple copies of Herpes simplex viral protein 16 domains (VP64) [25,26,30,48,49], the p65 domain [25], the combined VPR (VP64-p65-RTA) [50] and VTR3 [51] domains, the multimeric peptide array (SunTag) [27,52] and the synergistic activation mediator (SAM) [53,54] (Figure 1C).

To push the CRISPR technology forward, new CRISPR endonucleases besides Cas9 have been continuously excavated in the past decade. One subtype V endonuclease of the class 2 CRISPR system, Cas12a (previously known as Cpf1), has drawn more attention than others for its unique dual functionality to process its own pre-crRNA before cleaving dsDNA [61]. In the same way as Cas9, Cas12a has also been mutated to serve as transcription regulators. Multiple versions of DNase deactivated Cas12a (dCas12a), some of which have shown great potential for replacing dCas9 in genetic switches [31,32,33]. For example, dCas12a has been used alone or fused with KRAB or SRDX domains as CRISPRi, and functions as CRISPRa after fusing with VP64, p65 or VTR domain [31,32,33,34,35,36,38,55].

As with transcriptional factors, the CRISPR-based systems are also not perfect. The CRISPR/Cas9 system has been troubled by (1) the toxicity from off-target effects [62,63,64], (2) the large size of Cas9/dCas9 and (3) the lack of non-linearity [65,66]. First, the toxicity of Cas9 has severely limited its application potential, particularly in gene therapy where safety is the top concern [67]. Meanwhile, in a complex circuit with multiple dCas9-based switches, the off-target binding of dCas9 could also affect the orthogonality among these genetic switches. Second, the relatively large size of dCas9 makes it easy to cause physiological burden to the host, which limits its use in instances that require a high expression level of dCas9. For example, the limitation of dCas9 expression level could cap the maximum activation signal in CRISPRa or increase the background signal in CRISPRi, both conditions that pose a negative impact on the performance of dCas9-based genetic switches by reducing their dynamic range. Notably, the search for endonuclease smaller than Cas9 has led to the discovery of more compact endonucleases, including Cas12a [61], Cas12b [68], CasΦ [69] and CasX [70], which greatly promoted the delivery of CRISPR endonuclease in gene therapy. Third, the lack of non-linear behavior (e.g., ultrasensitivity) of CRISPR-based genetic switches is determined by the binding mechanism of CRISPR endonuclease, which functions by a monomeric endonuclease binding to DNA via a single binding site [65]. Achieving non-linear functions with CRISPR-based systems requires the cooperative activity of multimers or multiple binding sites [71], as in the TF-based genetic switches [72,73]. Therefore, to achieve non-linear functions with CRISPR-based switches, a more complex design is required.

## 3. CRISPR-Based Genetic Switches in Translation Level

Besides DNA endonuclease, CRISPR systems also contain endoribonuclease that recognize specific RNA sequences and structures on the repeats of pre-crRNA and cleaves at a certain location [74]. The genetic switches designed based on these endoribonucleases operate at the level of translation, which can work alongside the switches at the transcription level in genetic circuits [75].

**Figure 2:**
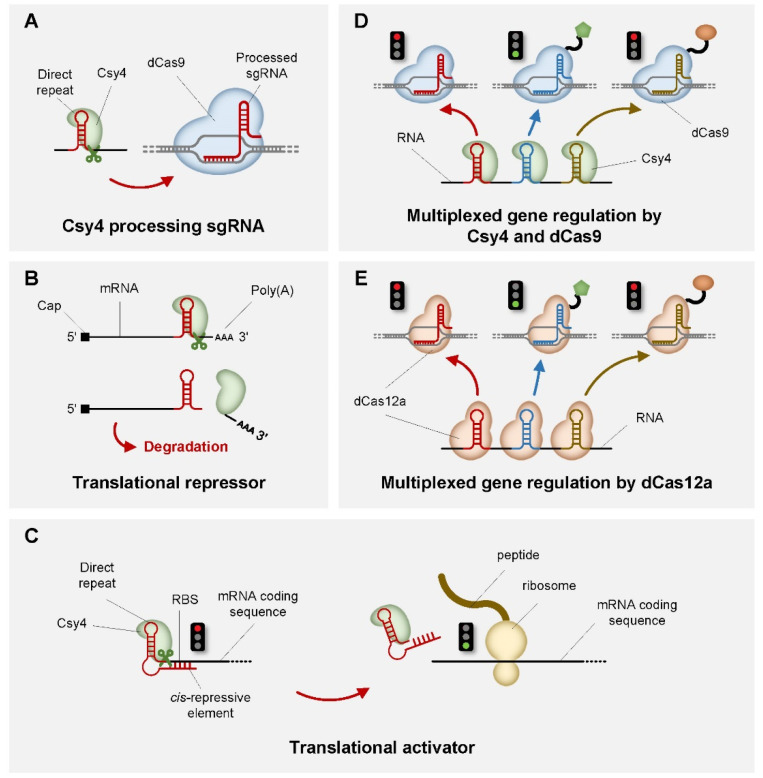
Schematic representation of CRISPR-based genetic switches in translational level. (**A**) Csy4 cleavage of the RNA transcript in the processing of gRNA. (**B**) Csy4 function as a translational repressor by decreasing mRNA stability through cleavage of the 3′ end. (**C**) Csy4 function as a translational activator. (**D**) Csy4 and dCas9 in multiplexed gene regulation. Different color of sgRNA indicates different sgRNA that directs dCas9 to the corresponding target genes. (**E**) Multiplexed gene regulation based on the dual functionality of Cas12a. Different color of sgRNA indicates different sgRNA that directs dCas12a to the corresponding target genes. Red light indicates repression, green light indicates activation.

The first widely used CRISPR endoribonuclease in a genetic switch is Csy4, a type I CRISPR endoribonuclease discovered in *Pseudomonas aeruginosa* that recognizes a 28-nt RNA hairpin structure and precisely cleaves between the 20G and 21C [76,77] (Figure 2A). Such precision enables Csy4 to control translation via cleaving of its target RNA hairpin inserted at the 5′ capping or 3′ UTR region of an mRNA, which could severely reduce mRNA stability and, consequently, translation rate [39] (Figure 2B). Furthermore, Csy4 has been demonstrated to control translation in a more sophisticated way for better performance and programmability. For example, a Csy4-based translational activator has been engineered by inserting the Csy4 target RNA hairpin between an RBS (Ribosome Binding Site) and a cis-repressive element [56]. As a result, mRNA translation is inhibited as the cis-repressive element binds to RBS in a complementary manner, and will be restarted after Csy4 cleavage that releases the cis-repressive element from the RBS (Figure 2C). As an endoribonuclease, Csy4 has also been used in many multiplexed gene regulations at translation level in yeast and mammalian cells [40,41,42,43]. By simultaneously processing multiple gRNAs with different target sequences for directing dCas9, Csy4, along with other similar endoribonuclease such as Cas5d, Cas6a and Cse3, can build complex logic gates for precisely regulating gene expression [56] (Figure 2D). Moreover, Cas12a, with the dual functionality of cleaving dsDNA and processing its own gRNA, is also valuable in multiplexed gene regulation because it allows simultaneous control of multiple genes using a single enzyme pairing with different gRNAs, which is much simpler than the Csy4/dCas9 systems [34,35,36,37,55] (Figure 2E).

Besides RNA endoribonuclease, another category of CRISPR enzyme, the RNA-guided RNA endonuclease, have also emerged in recent years with the potential as genetic switches at translational level, namely the Cas13a and Cas7-11 endonuclease [78,79,80]. These enzymes possess two distinct RNase activities: (1) processing their own pre-crRNA in a way similar to Csy4 [81]; (2) targeting specific RNA sequence for cleavage. Such dual functionality makes them particularly ideal for the knockdown of RNA transcripts in vivo. Specifically, Cas13a, a subtype VI effector in class 2 CRISPR-Cas systems, previously known as C2c2, has been used for gene silencing in *E. coli*, plants and human cells [80,82]. Cas13a possesses a unique “collateral” cleavage effect, which means, upon recognition of its target, Cas13a engages cleavage of nearby non-target RNA [79]. Such a cleavage pattern of Cas13a has been harnessed in a nucleic acid detection method known as SHERLOCK (Specific High Sensitivity Enzymatic Reporter UnLOCKing), in which the target nucleotide sequence is incorporated in the gRNA, and an RNA probe is designed to be fluorescent after being cleaved via “collateral” cleavage [83]. Cas7-11, on the other hand, is a subtype III effector in the class 1 CRISPR-Cas system recently reported to have a similar function as Cas13a [78]. Currently, although these endonucleases are primarily being employed for gene silencing, it is apparent that they have great potential to be engineered as multiplexed genetic switches similar to Csy4 and Cas12a.

## 4. Application of CRISPR-Based Switches in Genetic Circuits

With the individual genetic switches, larger circuits can be built to offer more sophisticated functions and provide greater potential for a wider range of applications. Thus far, a number of CRISPR-based genetic circuits have been reported, which primarily incorporate CRISPR-based genetic switches in three different ways: (1) Ligand/Light-inducible genetic switches, (2) genetic switches with certain non-linear behavior or feedback patterns, (3) multi-input biocomputation circuits (Table 2). The first refers to CRISPR-based genetic switches capable of sensing input signals (e.g., chemical ligands) and generates certain output signals; the second mainly includes the circuits that still function as a genetic switch, but offer non-linear functions such as ultrasensitivity, biostability, oscillation or IFFL (incoherent feed-forward loop); the third implies the circuits capable of computing Boolean logics including AND, NOT, NOR and XOR, etc.

**Figure 3:**
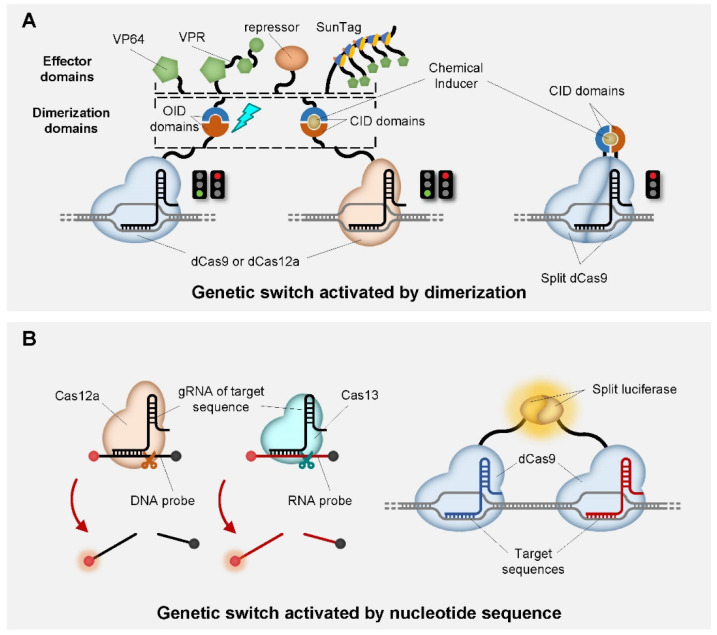
Schematic representation of genetic switches inducible by light, chemical ligands and nucleotide sequences. (**A**) Genetic switches activated by light- (left) or chemical ligand- (middle) induced dimerization that connects CRISPR endonuclease (dCas9 or dCas12) and activation/repression domain, as well as dimerization of split dCas9 induced by chemical inducer (right). Activation or repression depends on the type of the domains recruited after dimerization. Red light indicates repression, green light indicates activation. (**B**) Genetic switches activated by nucleotide sequences identified by Cas12a (left), Cas13 (middle) and dCas9 (right). Glows indicate fluorescence on probes (left and middle), or luminescence stimulated by luciferase.

Ligand/Light-inducible genetic switches are powerful tools for understanding the spatial and temporal pattern of gene expression because they offer the crucial capability of controlling genetic switches with external or internal input signals, such as chemicals or the light of specific wavelengths (Figure 3A, left and middle). Typically, such inducibility is achieved by endonuclease (mainly dCas9) fused with chemical-induced dimerizing domains (CID) or optogenetically inducible dimerizing domains (OID) that sense chemicals or light signals, respectively. Specifically, the two parts of dimerization domains are separately fused with endonucleases (dCas9 or dCas12a) and the effector domain. Then, dimerization occurs when certain chemical ligands or lights of certain wavelengths are detected, which completes the recruitment of the effector domain to the endonuclease binding sites on DNA. Thus far, the reported CIDs include FKBP-FRB [84,85] and DmrA-DmrC [55] induced by rapamycin, ABI-PYL1 induced by abscisic acid (ABA) [84,86] and GID1-GAI24 induced by gibberellin (GA) [86]. Meanwhile, various OIDs have also been reported, including PhyB-PIF induced by red light [87], and pMag-nMag and CRY2-CIB1 induced by blue light [88,89,90,91]. Alternatively, genetic switches can also be constructed based on split dCas9 (Figure 3A, right). In this case, dCas9 is split into two pieces and each piece is fused with part of a heterodimer [53,85,89]. Upon ligand induction, heterodimerization leads to the restoration of dCas9 enzymatic activity and subsequent DNA binding, which allows dCas9 to act as a transcriptional repressor [85]. Similarly, Cas9 can also be split and restored as an inducible genome editor [89].

Besides chemicals and light signals, oligonucleotide sequences can also be a form of input signal that activates inducible CRISPR-based genetic switches. In other words, CRISPR endonuclease (e.g., dCas9 or dCasd12a) recognizes specific DNA or RNA sequences as input signals, and activates subsequent signals accordingly. Such switches are particularly suitable for nucleotide detection, which makes them quite popular as IVD (in vitro diagnostic) methods. For example, several toolkits for detecting SARS-CoV-2 have been developed based on Cas12a or Cas13a, which functions by detecting specific RNA and cleaving complementary DNA or RNA probes as output signals, respectively [92,93,94,95,96,97] (Figure 3B, left). Additionally, IVD methods using dCas9 have also been reported, in which the two parts of a split luciferase are fused with two dCas9 molecules. Positive signals are generated when the enzymatic activity of luciferase is restored upon proper placement of the two dCas9 on the target DNA [98] (Figure 3B, right).

**Figure 4:**
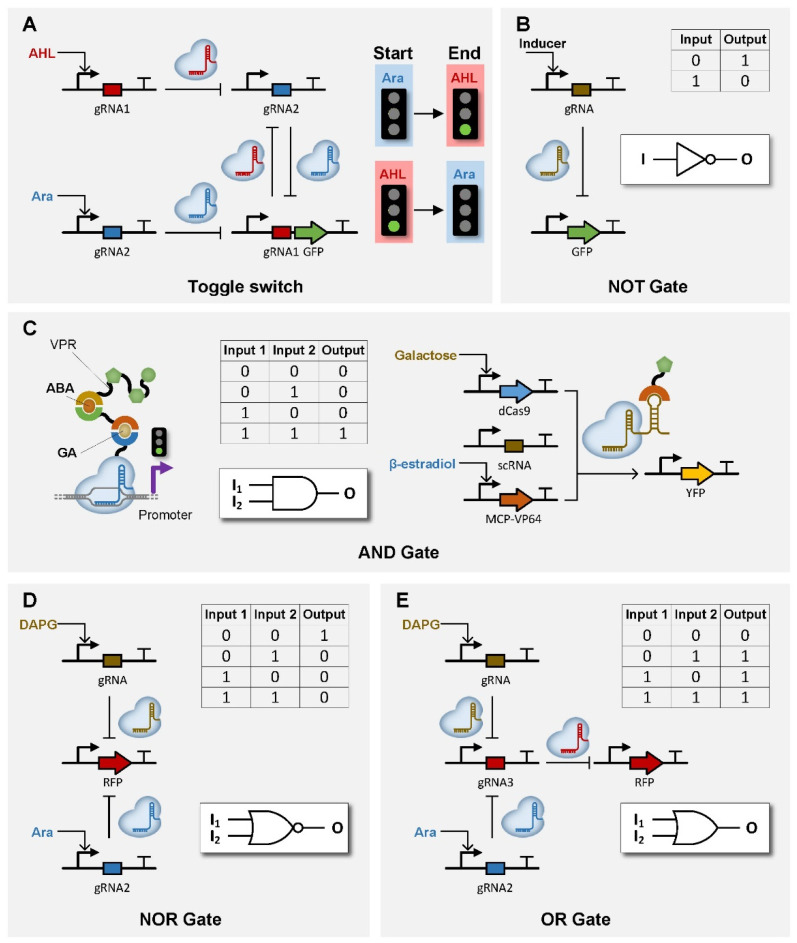
Schematic representation of CRISPR-based non-linear and Boolean logic circuits. (**A**) Toggle switch built by dCas9 with two different gRNA controlled by two different input signals. (**B**–**E**) Boolean logic circuits including NOT gate (**B**), AND gate (**C**), NOR gate (**D**) and OR gate (**E**).

As described above, one of the problems of CRISPR-based genetic switches is the lack of non-linear function. However, such a shortcoming can be overcome with a more complex design of CRISPR-based circuits that offers ultrasensitive, bistable and oscillatory signals. For example, toggle switches with ultrasensitive and bistable signals have been built with dCas9 in *E. coli* that achieved bistable toggle between two states [99] (Figure 4A). Moreover, oscillators based on Cas9 and Cas12a have also emerged recently with a robust oscillatory state in microfluid chambers [99,100,101]. Additionally, circuits that exhibit spatial-temporal behaviors can also be constructed with CRISPR-based genetic switches. By combining CRISPRi and CRISPRa, IFFL circuits have been constructed in *E. coli* and mammalian cells that display pulse-generating and stripe-forming patterns [99,102,103].

Biocomputation with logic gates is an important task of genetic circuits for signal integration, processing and logic computation. The foundation of biocomputation is various types of logic gates with robust performance. Many Boolean logic gates, such as AND, NOT and NOR gates, have been constructed with CRISPR-based circuits that functions in *E. coli*, yeast and human cells. For example, one-input logic gate, such as a NOT gate, can simply be a dCas9-based repressor, which converts “1” to “0” (Figure 4B). Likewise, more layer of repressors (or activators) can be connected in a consecutive manner, which will further convert the signal back to “1” and then jump back and forth between “0” and “1” [65,66,99,104,106,107,108,109]. Thus far, the input signal can be converted up to seven times with NOT gates [104]. On the other hand, the circuit designs to integrate two signals with a single logic gate are more complex and diverse. A straightforward design strategy is to control the expression of two orthogonal sgRNAs and their corresponding endonucleases with two different inducers that serve as input signals [65]. In this case, the type of logic gate is determined by the design of the two endonucleases. Specifically, two transcriptional activators will make the circuit an AND gate [105] (Figure 4C, right), while two transcriptional repressors will result in a NOR gate [65,104] (Figure 4D). Alternatively, an AND gate can be built with other methods such as heterodimerization induced by two different ligands [53,85,86,89] (Figure 4C, left). Notably, a NOR gate is known to be “Boolean-complete”, which means all types of logic gates can be built by combining NOR gates in different ways. For example, converting the output signal of a NOR gate with a NOT gate creates an OR gate [65] (Figure 4E). Similarly, by converting the two input signals of a NOR gate with two NOT gates beforehand, the combination of the three gates (two NOT gates and one NOR gate) equals to an AND gate [65].

## 5. Conclusions

The enzymes discovered from CRISPR systems, with their abundance, diversity and unrivaled programmability, have shown remarkable value and potential in the construction of genetic switches operating in both transcription and translation levels. The prevalence of their existence in nature has also been a great gift that has bestowed Cas9, Cas12a, Cas13, Csy4, and Cas7-11 and much more to come. The advantages of genetic switches constructed with these enzymes have attracted the interest of many circuit designers who have reported a number of circuits with diverse functions in return. It is apparent that CRISPR-based genetic switches will continue to stay in the spotlight of the research of synthetic biology and make their potential into reality in various applications.

## Figures and Tables

**Table 1:** CRISPR-based genetic switches.

CRISPR Enzyme	Additional Module	Function	Host	Reference
dCas9	/	Transcriptional repression	bacteria, yeast	[23,24,25,26]
dCas9	KRAB (F)	Transcriptional repression	yeast, mammalian cell	[25,26,27,28,29,30]
dCas9	Mxi1 (F)	Transcriptional repression	yeast	[26]
dCas9	SRDX (F)	Transcriptional repression	*Arabidopsis*	[30]
dCas12a	/	Transcriptional repression	bacteria, mammalian cell	[31,32,33,34,35]
dCas12a	KRAB (F)	Transcriptional repression	mammalian cell	[33,36]
dCas12a	Mxi1 (F)	Transcriptional repression	yeast	[37]
dCas12a	SRDX (F)	Transcriptional repression	*Arabidopsis*	[38]
Csy4	/	Translational repression	*E. coli*, mammalian cell	[39]
Csy4	dCas9	Translational repression	mammalian cell	[40]
Csy4	dCas9	Translational repression	yeast, mammalian cell	[41]
Csy4	dCas9	Translational repression	yeast	[42]
Csy4	dCas9/dCas12a	Translational repression	yeast	[43]
dCas9	RNAP ω subunit (F)	Transcriptional activation	*E. coli*	[24]
dCas9	AsiA (F)	Transcriptional activation	*E. coli*	[44]
dCas9	MCP-SoxS (R)	Transcriptional activation	*E. coli*	[45,46]
dCas9	λN22plus-PspFΔHTH (R)	Transcriptional activation	*E. coli*	[47]
dCas9	VP64 (F)	Transcriptional activation	yeast, mammalian cell, *Arabidopsis*	[25,26,30,48,49]
dCas9	p65 (F)	Transcriptional activation	mammalian cell	[25]
dCas9	VPR (F)	Transcriptional activation	yeast, mammalian cell	[50]
dCas9	VTR3 (F)	Transcriptional activation	mammalian cell	[51]
dCas9	SunTag (F)	Transcriptional activation	mammalian cell	[27,52]
dCas9	SAM (F)	Transcriptional activation	*E. coli*, mammalian cell	[53,54]
dCas12a	VP64 (F)	Transcriptional activation	mammalian cell	[33]
dCas12a	p65 (F)	Transcriptional activation	mammalian cell	[55]
dCas12a	VPR (F)	Transcriptional activation	mammalian cell	[33]
Csy4	/	Translational activation	*E. coli*	[56]
Csy4	Cas9	Translational activation	yeast, mammalian cell	[41]

(F) indicate module directly fused to CRISPR enzyme; (R) indicate module recruited by CRISPR enzyme or its associated gRNA.

**Table 2:** Applications of CRISPR-based genetic switches in circuits.

CRISPR Enzyme	Dimerization Domain	Effector Domain	Input Signal	Circuit Type	Reference
dCas9	FKBP-FRB	VPR	rapamycin	ligand-inducible genetic switch	[84]
Split dCas9	FKBP-FRB	VP64	rapamycin	ligand-inducible genetic switch	[85]
dCas12a	DmrA-DmrC	p65, VPR	rapamycin	ligand-inducible genetic switch	[55]
dCas9	ABI-PYL1	VPR	ABA	ligand-inducible genetic switch	[84,86]
dCas9	GID1-GAI24	VPR	GA	ligand-inducible genetic switch	[86]
dCas9	PhyB-PIF	/	Red light	Light-inducible genetic switch	[87]
dCas9	pMag-nMag	p65, VP64	Blue light	Light-inducible genetic switch	[88]
Split Cas9	pMag-nMag	/	Blue light	Light-inducible genome editor	[89]
dCas9	CRY2-CIB1	p65, VP64	Blue light	Light-inducible genetic switch	[88,89,90,91]
Cas12a	/	fluorescent DNA probe	nucleotide sequence	in vitro diagnostic toolbox	[92,93,94,95]
Cas13	/	fluorescent RNA probe	nucleotide sequence	in vitro diagnostic toolbox	[96,97]
dCas9	split luciferease	luciferase	nucleotide sequence	in vitro diagnostic toolbox	[98]
dCas9	/	/	AHL & Ara	toggle switch	[99]
dCas9	/	/	/	oscillator	[99,100,101]
dCas9	/	/	Ara	IFFL	[99]
CasE	/	VPR	DNA copy number	IFFL	[102]
dCas9	/	KRAB, VPR	Anti-CRISPR protein	IFFL	[103]
dCas9	/	/	DAPG and Ara	NOR/AND/OR gate	[65]
dCas9	/	Mxi1	gRNA	NOR gateNOT gate	[104]
dCas9	GID1-GAI24	VPR	GA and ABA	AND gate	[86]
dCas9		VP64	Galactose and β-estradiol	AND gate	[105]
dCas9	/	/	Ara	NOT gate	[99,106]
dCas9	/	VP16	gRNA	NOT gate	[107]
dCas9	/	/	aTc	NOT gate	[108]
dCas9	/	KRAB	gRNA	NOT gate	[109]

**GA**: gibberellin; **ABA**: abscisic acid; **DAPG**: 2,4-Diacetylphloroglucinol; **Ara**: Arabinose; **AHL**: acyl homoserine lactone; **aTc**: Anhydrotetracycline.

## Data Availability

Not applicable.

## References

[1] Gardner T.S., Cantor C.R., Collins J.J. (2000). Construction of a genetic toggle switch in Escherichia coli. Nature.

[2] Prindle A., Samayoa P., Razinkov I., Danino T., Tsimring L.S., Hasty J. (2011). A sensing array of radically coupled genetic ‘biopixels’. Nature.

[3] Liu C., Fu X., Liu L., Ren X., Chau C.K., Li S., Xiang L., Zeng H., Chen G., Tang L.H. (2011). Sequential establishment of stripe patterns in an expanding cell population. Science.

[4] Schaerli Y., Munteanu A., Gili M., Cotterell J., Sharpe J., Isalan M. (2014). A unified design space of synthetic stripe-forming networks. Nat. Commun..

[5] Tamsir A., Tabor J.J., Voigt C.A. (2011). Robust multicellular computing using genetically encoded NOR gates and chemical ‘wires’. Nature.

[6] Auslander D., Auslander S., Pierrat X., Hellmann L., Rachid L., Fussenegger M. (2018). Programmable full-adder computations in communicating three-dimensional cell cultures. Nat. Methods.

[7] Stanton B.C., Nielsen A.A., Tamsir A., Clancy K., Peterson T., Voigt C.A. (2014). Genomic mining of prokaryotic repressors for orthogonal logic gates. Nat. Chem. Biol..

[8] Farzadfard F., Lu T.K. (2014). Synthetic biology. Genomically encoded analog memory with precise in vivo DNA writing in living cell populations. Science.

[9] Bonnet J., Subsoontorn P., Endy D. (2012). Rewritable digital data storage in live cells via engineered control of recombination directionality. Proc. Natl. Acad. Sci. USA.

[10] Du P., Zhao H., Zhang H., Wang R., Huang J., Tian Y., Luo X., Luo X., Wang M., Xiang Y. (2020). De novo design of an intercellular signaling toolbox for multi-channel cell-cell communication and biological computation. Nat. Commun..

[11] Billerbeck S., Brisbois J., Agmon N., Jimenez M., Temple J., Shen M., Boeke J.D., Cornish V.W. (2018). A scalable peptide-GPCR language for engineering multicellular communication. Nat. Commun..

[12] Bacchus W., Lang M., El-Baba M.D., Weber W., Stelling J., Fussenegger M. (2012). Synthetic two-way communication between mammalian cells. Nat. Biotechnol..

[13] Chen M.T., Weiss R. (2005). Artificial cell-cell communication in yeast Saccharomyces cerevisiae using signaling elements from Arabidopsis thaliana. Nat. Biotechnol..

[14] Gupta A., Reizman I.M., Reisch C.R., Prather K.L. (2017). Dynamic regulation of metabolic flux in engineered bacteria using a pathway-independent quorum-sensing circuit. Nat. Biotechnol..

[15] Soma Y., Hanai T. (2015). Self-induced metabolic state switching by a tunable cell density sensor for microbial isopropanol production. Metab. Eng..

[16] Tsao C.Y., Hooshangi S., Wu H.C., Valdes J.J., Bentley W.E. (2010). Autonomous induction of recombinant proteins by minimally rewiring native quorum sensing regulon of E. coli. Metab. Eng..

[17] Stephens K., Pozo M., Tsao C.Y., Hauk P., Bentley W.E. (2019). Bacterial co-culture with cell signaling translator and growth controller modules for autonomously regulated culture composition. Nat. Commun..

[18] Kitada T., DiAndreth B., Teague B., Weiss R. (2018). Programming gene and engineered-cell therapies with synthetic biology. Science.

[19] Sedlmayer F., Jaeger T., Jenal U., Fussenegger M. (2017). Quorum-Quenching Human Designer Cells for Closed-Loop Control of Pseudomonas aeruginosa Biofilms. Nano Lett..

[20] Saeidi N., Wong C.K., Lo T.M., Nguyen H.X., Ling H., Leong S.S., Poh C.L., Chang M.W. (2011). Engineering microbes to sense and eradicate Pseudomonas aeruginosa, a human pathogen. Mol. Syst. Biol..

[21] Sorek R., Lawrence C.M., Wiedenheft B. (2013). CRISPR-mediated adaptive immune systems in bacteria and archaea. Annu Rev. Biochem..

[22] Makarova K.S., Haft D.H., Barrangou R., Brouns S.J., Charpentier E., Horvath P., Moineau S., Mojica F.J., Wolf Y.I., Yakunin A.F. (2011). Evolution and classification of the CRISPR-Cas systems. Nat. Rev. Microbiol..

[23] Qi L.S., Larson M.H., Gilbert L.A., Doudna J.A., Weissman J.S., Arkin A.P., Lim W.A. (2013). Repurposing CRISPR as an RNA-guided platform for sequence-specific control of gene expression. Cell.

[24] Bikard D., Jiang W., Samai P., Hochschild A., Zhang F., Marraffini L.A. (2013). Programmable repression and activation of bacterial gene expression using an engineered CRISPR-Cas system. Nucleic Acids Res..

[25] Farzadfard F., Perli S.D., Lu T.K. (2013). Tunable and multifunctional eukaryotic transcription factors based on CRISPR/Cas. ACS Synth. Biol..

[26] Gilbert L.A., Larson M.H., Morsut L., Liu Z., Brar G.A., Torres S.E., Stern-Ginossar N., Brandman O., Whitehead E.H., Doudna J.A. (2013). CRISPR-mediated modular RNA-guided regulation of transcription in eukaryotes. Cell.

[27] Gilbert L.A., Horlbeck M.A., Adamson B., Villalta J.E., Chen Y., Whitehead E.H., Guimaraes C., Panning B., Ploegh H.L., Bassik M.C. (2014). Genome-Scale CRISPR-Mediated Control of Gene Repression and Activation. Cell.

[28] Thakore P.I., D’Ippolito A.M., Song L., Safi A., Shivakumar N.K., Kabadi A.M., Reddy T.E., Crawford G.E., Gersbach C.A. (2015). Highly specific epigenome editing by CRISPR-Cas9 repressors for silencing of distal regulatory elements. Nat. Methods.

[29] Mandegar M.A., Huebsch N., Frolov E.B., Shin E., Truong A., Olvera M.P., Chan A.H., Miyaoka Y., Holmes K., Spencer C.I. (2016). CRISPR Interference Efficiently Induces Specific and Reversible Gene Silencing in Human iPSCs. Cell Stem Cell.

[30] Lowder L.G., Zhang D., Baltes N.J., Paul J.W., Tang X., Zheng X., Voytas D.F., Hsieh T.F., Zhang Y., Qi Y. (2015). A CRISPR/Cas9 Toolbox for Multiplexed Plant Genome Editing and Transcriptional Regulation. Plant. Physiol..

[31] Miao C., Zhao H., Qian L., Lou C. (2019). Systematically investigating the key features of the DNase deactivated Cpf1 for tunable transcription regulation in prokaryotic cells. Synth. Syst. Biotechnol..

[32] Kim S.K., Kim S.H., Subhadra B., Woo S.G., Rha E., Kim S.W., Kim H., Lee D.H., Lee S.G. (2018). A Genetically Encoded Biosensor for Monitoring Isoprene Production in Engineered Escherichia coli. ACS Synth. Biol..

[33] Liu Y., Han J., Chen Z., Wu H., Dong H., Nie G. (2017). Engineering cell signaling using tunable CRISPR-Cpf1-based transcription factors. Nat. Commun..

[34] Li M., Chen J., Wang Y., Liu J., Huang J., Chen N., Zheng P., Sun J. (2020). Efficient Multiplex Gene Repression by CRISPR-dCpf1 in Corynebacterium glutamicum. Front. Bioeng. Biotechnol..

[35] Campa C.C., Weisbach N.R., Santinha A.J., Incarnato D., Platt R.J. (2019). Multiplexed genome engineering by Cas12a and CRISPR arrays encoded on single transcripts. Nat. Methods.

[36] Zhang J.L., Peng Y.Z., Liu D., Liu H., Cao Y.X., Li B.Z., Li C., Yuan Y.J. (2018). Gene repression via multiplex gRNA strategy in Y. lipolytica. Microb. Cell Fact..

[37] Ciurkot K., Gorochowski T.E., Roubos J.A., Verwaal R. (2021). Efficient multiplexed gene regulation in Saccharomyces cerevisiae using dCas12a. Nucleic Acids Res..

[38] Tang X., Lowder L.G., Zhang T., Malzahn A.A., Zheng X., Voytas D.F., Zhong Z., Chen Y., Ren Q., Li Q. (2017). A CRISPR-Cpf1 system for efficient genome editing and transcriptional repression in plants. Nat. Plants.

[39] Borchardt E.K., Vandoros L.A., Huang M., Lackey P.E., Marzluff W.F., Asokan A. (2015). Controlling mRNA stability and translation with the CRISPR endoribonuclease Csy4. RNA.

[40] Nissim L., Perli S.D., Fridkin A., Perez-Pinera P., Lu T.K. (2014). Multiplexed and programmable regulation of gene networks with an integrated RNA and CRISPR/Cas toolkit in human cells. Mol. Cell.

[41] Zalatan J.G., Lee M.E., Almeida R., Gilbert L.A., Whitehead E.H., La Russa M., Tsai J.C., Weissman J.S., Dueber J.E., Qi L.S. (2015). Engineering complex synthetic transcriptional programs with CRISPR RNA scaffolds. Cell.

[42] McCarty N.S., Shaw W.M., Ellis T., Ledesma-Amaro R. (2019). Rapid Assembly of gRNA Arrays via Modular Cloning in Yeast. ACS Synth. Biol..

[43] Lian J., HamediRad M., Hu S., Zhao H. (2017). Combinatorial metabolic engineering using an orthogonal tri-functional CRISPR system. Nat. Commun..

[44] Ho H.I., Fang J.R., Cheung J., Wang H.H. (2020). Programmable CRISPR-Cas transcriptional activation in bacteria. Mol. Syst. Biol..

[45] Dong C., Fontana J., Patel A., Carothers J.M., Zalatan J.G. (2018). Synthetic CRISPR-Cas gene activators for transcriptional reprogramming in bacteria. Nat. Commun..

[46] Fontana J., Dong C., Kiattisewee C., Chavali V.P., Tickman B.I., Carothers J.M., Zalatan J.G. (2020). Effective CRISPRa-mediated control of gene expression in bacteria must overcome strict target site requirements. Nat. Commun..

[47] Liu Y., Wan X., Wang B. (2019). Engineered CRISPRa enables programmable eukaryote-like gene activation in bacteria. Nat. Commun..

[48] Perez-Pinera P., Kocak D.D., Vockley C.M., Adler A.F., Kabadi A.M., Polstein L.R., Thakore P.I., Glass K.A., Ousterout D.G., Leong K.W. (2013). RNA-guided gene activation by CRISPR-Cas9-based transcription factors. Nat. Methods.

[49] Maeder M.L., Linder S.J., Cascio V.M., Fu Y., Ho Q.H., Joung J.K. (2013). CRISPR RNA-guided activation of endogenous human genes. Nat. Methods.

[50] Chavez A., Scheiman J., Vora S., Pruitt B.W., Tuttle M., Iyer E.P., Lin S., Kiani S., Guzman C.D., Wiegand D.J. (2015). Highly efficient Cas9-mediated transcriptional programming. Nat. Methods.

[51] Ma D., Peng S., Huang W., Cai Z., Xie Z. (2018). Rational Design of Mini-Cas9 for Transcriptional Activation. ACS Synth. Biol..

[52] Tanenbaum M.E., Gilbert L.A., Qi L.S., Weissman J.S., Vale R.D. (2014). A protein-tagging system for signal amplification in gene expression and fluorescence imaging. Cell.

[53] Baeumler T.A., Ahmed A.A., Fulga T.A. (2017). Engineering Synthetic Signaling Pathways with Programmable dCas9-Based Chimeric Receptors. Cell Rep..

[54] Konermann S., Brigham M.D., Trevino A.E., Joung J., Abudayyeh O.O., Barcena C., Hsu P.D., Habib N., Gootenberg J.S., Nishimasu H. (2015). Genome-scale transcriptional activation by an engineered CRISPR-Cas9 complex. Nature.

[55] Tak Y.E., Kleinstiver B.P., Nunez J.K., Hsu J.Y., Horng J.E., Gong J., Weissman J.S., Joung J.K. (2017). Inducible and multiplex gene regulation using CRISPR-Cpf1-based transcription factors. Nat. Methods.

[56] Du P., Miao C., Lou Q., Wang Z., Lou C. (2016). Engineering Translational Activators with CRISPR-Cas System. ACS Synth. Biol..

[57] Hsu P.D., Scott D.A., Weinstein J.A., Ran F.A., Konermann S., Agarwala V., Li Y., Fine E.J., Wu X., Shalem O. (2013). DNA targeting specificity of RNA-guided Cas9 nucleases. Nat. Biotechnol..

[58] Deltcheva E., Chylinski K., Sharma C.M., Gonzales K., Chao Y., Pirzada Z.A., Eckert M.R., Vogel J., Charpentier E. (2011). CRISPR RNA maturation by trans-encoded small RNA and host factor RNase III. Nature.

[59] Jinek M., Chylinski K., Fonfara I., Hauer M., Doudna J.A., Charpentier E. (2012). A programmable dual-RNA-guided DNA endonuclease in adaptive bacterial immunity. Science.

[60] Fu Y., Sander J.D., Reyon D., Cascio V.M., Joung J.K. (2014). Improving CRISPR-Cas nuclease specificity using truncated guide RNAs. Nat. Biotechnol..

[61] Chen J.S., Ma E., Harrington L.B., Da Costa M., Tian X., Palefsky J.M., Doudna J.A. (2018). CRISPR-Cas12a target binding unleashes indiscriminate single-stranded DNase activity. Science.

[62] Zhang X.H., Tee L.Y., Wang X.G., Huang Q.S., Yang S.H. (2015). Off-target Effects in CRISPR/Cas9-mediated Genome Engineering. Mol. Ther. Nucleic Acids.

[63] Fu Y., Foden J.A., Khayter C., Maeder M.L., Reyon D., Joung J.K., Sander J.D. (2013). High-frequency off-target mutagenesis induced by CRISPR-Cas nucleases in human cells. Nat. Biotechnol..

[64] Pattanayak V., Lin S., Guilinger J.P., Ma E., Doudna J.A., Liu D.R. (2013). High-throughput profiling of off-target DNA cleavage reveals RNA-programmed Cas9 nuclease specificity. Nat. Biotechnol..

[65] Nielsen A.A., Voigt C.A. (2014). Multi-input CRISPR/Cas genetic circuits that interface host regulatory networks. Mol. Syst. Biol..

[66] Menn D.J., Pradhan S., Kiani S., Wang X. (2018). Fluorescent Guide RNAs Facilitate Development of Layered Pol II-Driven CRISPR Circuits. ACS Synth. Biol..

[67] Uddin F., Rudin C.M., Sen T. (2020). CRISPR Gene Therapy: Applications, Limitations, and Implications for the Future. Front. Oncol..

[68] Teng F., Cui T., Feng G., Guo L., Xu K., Gao Q., Li T., Li J., Zhou Q., Li W. (2018). Repurposing CRISPR-Cas12b for mammalian genome engineering. Cell Discov..

[69] Pausch P., Al-Shayeb B., Bisom-Rapp E., Tsuchida C.A., Li Z., Cress B.F., Knott G.J., Jacobsen S.E., Banfield J.F., Doudna J.A. (2020). CRISPR-CasPhi from huge phages is a hypercompact genome editor. Science.

[70] Liu J.J., Orlova N., Oakes B.L., Ma E., Spinner H.B., Baney K.L.M., Chuck J., Tan D., Knott G.J., Harrington L.B. (2019). CasX enzymes comprise a distinct family of RNA-guided genome editors. Nature.

[71] Ji X., Zhao H., Zhu H., Zhu K., Tang S.Y., Lou C. (2020). CRISPRi/dCpf1-mediated dynamic metabolic switch to enhance butenoic acid production in Escherichia coli. Appl. Microbiol. Biotechnol..

[72] Hou J., Zeng W., Zong Y., Chen Z., Miao C., Wang B., Lou C. (2018). Engineering the Ultrasensitive Transcription Factors by Fusing a Modular Oligomerization Domain. ACS Synth. Biol..

[73] Zeng W., Du P., Lou Q., Wu L., Zhang H.M., Lou C., Wang H., Ouyang Q. (2017). Rational Design of an Ultrasensitive Quorum-Sensing Switch. ACS Synth. Biol..

[74] Wright A.V., Nunez J.K., Doudna J.A. (2016). Biology and Applications of CRISPR Systems: Harnessing Nature’s Toolbox for Genome Engineering. Cell.

[75] Qi L., Haurwitz R.E., Shao W., Doudna J.A., Arkin A.P. (2012). RNA processing enables predictable programming of gene expression. Nat. Biotechnol..

[76] Haurwitz R.E., Jinek M., Wiedenheft B., Zhou K., Doudna J.A. (2010). Sequence- and structure-specific RNA processing by a CRISPR endonuclease. Science.

[77] Haurwitz R.E., Sternberg S.H., Doudna J.A. (2012). Csy4 relies on an unusual catalytic dyad to position and cleave CRISPR RNA. EMBO J..

[78] Ozcan A., Krajeski R., Ioannidi E., Lee B., Gardner A., Makarova K.S., Koonin E.V., Abudayyeh O.O., Gootenberg J.S. (2021). Programmable RNA targeting with the single-protein CRISPR effector Cas7-11. Nature.

[79] Abudayyeh O.O., Gootenberg J.S., Konermann S., Joung J., Slaymaker I.M., Cox D.B., Shmakov S., Makarova K.S., Semenova E., Minakhin L. (2016). C2c2 is a single-component programmable RNA-guided RNA-targeting CRISPR effector. Science.

[80] Abudayyeh O.O., Gootenberg J.S., Essletzbichler P., Han S., Joung J., Belanto J.J., Verdine V., Cox D.B.T., Kellner M.J., Regev A. (2017). RNA targeting with CRISPR-Cas13. Nature.

[81] East-Seletsky A., O’Connell M.R., Knight S.C., Burstein D., Cate J.H., Tjian R., Doudna J.A. (2016). Two distinct RNase activities of CRISPR-C2c2 enable guide-RNA processing and RNA detection. Nature.

[82] Liao C., Ttofali F., Slotkowski R.A., Denny S.R., Cecil T.D., Leenay R.T., Keung A.J., Beisel C.L. (2019). Modular one-pot assembly of CRISPR arrays enables library generation and reveals factors influencing crRNA biogenesis. Nat. Commun..

[83] Gootenberg J.S., Abudayyeh O.O., Lee J.W., Essletzbichler P., Dy A.J., Joung J., Verdine V., Donghia N., Daringer N.M., Freije C.A. (2017). Nucleic acid detection with CRISPR-Cas13a/C2c2. Science.

[84] Bao Z., Jain S., Jaroenpuntaruk V., Zhao H. (2017). Orthogonal Genetic Regulation in Human Cells Using Chemically Induced CRISPR/Cas9 Activators. ACS Synth. Biol..

[85] Zetsche B., Volz S.E., Zhang F. (2015). A split-Cas9 architecture for inducible genome editing and transcription modulation. Nat. Biotechnol..

[86] Gao Y., Xiong X., Wong S., Charles E.J., Lim W.A., Qi L.S. (2016). Complex transcriptional modulation with orthogonal and inducible dCas9 regulators. Nat. Methods.

[87] Levskaya A., Weiner O.D., Lim W.A., Voigt C.A. (2009). Spatiotemporal control of cell signalling using a light-switchable protein interaction. Nature.

[88] Nihongaki Y., Furuhata Y., Otabe T., Hasegawa S., Yoshimoto K., Sato M. (2017). CRISPR-Cas9-based photoactivatable transcription systems to induce neuronal differentiation. Nat. Methods.

[89] Nihongaki Y., Kawano F., Nakajima T., Sato M. (2015). Photoactivatable CRISPR-Cas9 for optogenetic genome editing. Nat. Biotechnol..

[90] Polstein L.R., Gersbach C.A. (2015). A light-inducible CRISPR-Cas9 system for control of endogenous gene activation. Nat. Chem. Biol..

[91] Nihongaki Y., Yamamoto S., Kawano F., Suzuki H., Sato M. (2015). CRISPR-Cas9-based photoactivatable transcription system. Chem. Biol..

[92] Broughton J.P., Deng X., Yu G., Fasching C.L., Servellita V., Singh J., Miao X., Streithorst J.A., Granados A., Sotomayor-Gonzalez A. (2020). CRISPR-Cas12-based detection of SARS-CoV-2. Nat. Biotechnol..

[93] Ding X., Yin K., Li Z., Lalla R.V., Ballesteros E., Sfeir M.M., Liu C. (2020). Ultrasensitive and visual detection of SARS-CoV-2 using all-in-one dual CRISPR-Cas12a assay. Nat. Commun..

[94] Ooi K.H., Liu M.M., Tay J.W.D., Teo S.Y., Kaewsapsak P., Jin S., Lee C.K., Hou J., Maurer-Stroh S., Lin W. (2021). An engineered CRISPR-Cas12a variant and DNA-RNA hybrid guides enable robust and rapid COVID-19 testing. Nat. Commun..

[95] Xiong D., Dai W., Gong J., Li G., Liu N., Wu W., Pan J., Chen C., Jiao Y., Deng H. (2020). Rapid detection of SARS-CoV-2 with CRISPR-Cas12a. PLoS Biol..

[96] Fozouni P., Son S., Diaz de Leon Derby M., Knott G.J., Gray C.N., D’Ambrosio M.V., Zhao C., Switz N.A., Kumar G.R., Stephens S.I. (2021). Amplification-free detection of SARS-CoV-2 with CRISPR-Cas13a and mobile phone microscopy. Cell.

[97] Patchsung M., Jantarug K., Pattama A., Aphicho K., Suraritdechachai S., Meesawat P., Sappakhaw K., Leelahakorn N., Ruenkam T., Wongsatit T. (2020). Clinical validation of a Cas13-based assay for the detection of SARS-CoV-2 RNA. Nat. Biomed. Eng..

[98] Zhang Y., Qian L., Wei W., Wang Y., Wang B., Lin P., Liu W., Xu L., Li X., Liu D. (2017). Paired Design of dCas9 as a Systematic Platform for the Detection of Featured Nucleic Acid Sequences in Pathogenic Strains. ACS Synth. Biol..

[99] Santos-Moreno J., Tasiudi E., Stelling J., Schaerli Y. (2020). Multistable and dynamic CRISPRi-based synthetic circuits. Nat. Commun..

[100] Kuo J., Yuan R., Sanchez C., Paulsson J., Silver P.A. (2020). Toward a translationally independent RNA-based synthetic oscillator using deactivated CRISPR-Cas. Nucleic Acids Res..

[101] Henningsen J., Schwarz-Schilling M., Leibl A., Gutierrez J.N., Sagredo S., Simmel F.C. (2020). Single Cell Characterization of a Synthetic Bacterial Clock with a Hybrid Feedback Loop Containing dCas9-sgRNA. ACS Synth. Biol..

[102] Jones R.D., Qian Y., Siciliano V., DiAndreth B., Huh J., Weiss R., Del Vecchio D. (2020). An endoribonuclease-based feedforward controller for decoupling resource-limited genetic modules in mammalian cells. Nat. Commun..

[103] Nakamura M., Srinivasan P., Chavez M., Carter M.A., Dominguez A.A., La Russa M., Lau M.B., Abbott T.R., Xu X., Zhao D. (2019). Anti-CRISPR-mediated control of gene editing and synthetic circuits in eukaryotic cells. Nat. Commun..

[104] Gander M.W., Vrana J.D., Voje W.E., Carothers J.M., Klavins E. (2017). Digital logic circuits in yeast with CRISPR-dCas9 NOR gates. Nat. Commun..

[105] Hofmann A., Falk J., Prangemeier T., Happel D., Kober A., Christmann A., Koeppl H., Kolmar H. (2019). A tightly regulated and adjustable CRISPR-dCas9 based AND gate in yeast. Nucleic Acids Res..

[106] Santos-Moreno J., Schaerli Y. (2019). A Framework for the Modular and Combinatorial Assembly of Synthetic Gene Circuits. ACS Synth. Biol..

[107] Kiani S., Beal J., Ebrahimkhani M.R., Huh J., Hall R.N., Xie Z., Li Y., Weiss R. (2014). CRISPR transcriptional repression devices and layered circuits in mammalian cells. Nat. Methods.

[108] Didovyk A., Borek B., Hasty J., Tsimring L. (2016). Orthogonal Modular Gene Repression in Escherichia coli Using Engineered CRISPR/Cas9. ACS Synth. Biol..

[109] Kim H., Bojar D., Fussenegger M. (2019). A CRISPR/Cas9-based central processing unit to program complex logic computation in human cells. Proc. Natl. Acad. Sci. USA.

